# *COMT1*-dependent melatonin biosynthesis mediates *Trichoderma asperellum*-induced resistance to *Cladosporium fulvum* leaf mold in tomato

**DOI:** 10.3389/fpls.2026.1912163

**Published:** 2026-08-04

**Authors:** Yafei Yan, Zhiyu Song, Kejia Zhang, Ning Guo, Zhan Ma, Long Zhao, Golam Jalal Ahammed, Airong Liu, Shuangchen Chen

**Affiliations:** 1 College of Horticulture and Plant Protection, Henan University of Science and Technology, Luoyang, China; 2 Henan International Joint Laboratory of Stress Resistance Regulation and Safe Production of Protected Vegetables, Luoyang, China; 3 Henan Engineering Technology Research Center for Horticultural Crop Safety and Disease Control, Luoyang, China

**Keywords:** *Cladosporium fulvum*, COMT1, leaf mold, melatonin, *Solanum lycopersicum*, *Trichoderma*

## Abstract

Tomato leaf mold, caused by *Cladosporium fulvum*, is a destructive foliar disease in protected cultivation. Biological control using *Trichoderma* and plant defense elicitors such as melatonin offers a sustainable alternative to chemical fungicides. However, the synergistic effect of combining *Trichoderma* with melatonin and the role of endogenous melatonin in *Trichoderma*-induced resistance to tomato leaf mold remain unclear. In this study, we screened ten *Trichoderma* strains and identified *T. asperellum* T141 strain as the most effective antagonist against *C. fulvum* in dual culture assays. Moreover, exogenous melatonin (100 μmol/L) resulted in the lowest disease index and significantly reduced malondialdehyde content. The combined application of *T. asperellum* and melatonin prior to pathogen inoculation reduced the disease index by 77.57% and promoted plant growth compared with pathogen-only controls. The combination also decreased H_2_O_2_ and O_2_^·-^, elevated antioxidant enzyme (SOD, POD, CAT, and APX) activities, and restored photosynthetic parameters, pigment contents, Rubisco activity, FBPase activity, and expression of photosynthesis-related genes (*FBPase*, *SBPase*, *FBPA*, and *TPI*). Virus-induced gene silencing of the *COMT1* gene, a key melatonin biosynthesis gene, drastically reduced endogenous melatonin, and largely compromised *T. asperellum*-induced resistance, along with attenuated antioxidant defense and photosynthetic recovery. Collectively, our results demonstrate that *T. asperellum* and melatonin synergistically protect tomato against *C. fulvum* by mitigating oxidative stress and preserving photosynthetic function, and that *COMT1*-dependent endogenous melatonin synthesis is essential for *T. asperellum*-induced resistance. This study provides a theoretical basis for developing *Trichoderma*-melatonin biopreparations as an eco-friendly strategy for the management of tomato leaf mold.

## 1 Introduction

Tomato (*Solanum lycopersicum* L.) is one of the most widely cultivated vegetable crops worldwide, but its production is constantly threatened by various fungal diseases (Gunasekaran et al., 2026). Among them, tomato leaf mold, caused by the biotrophic fungus *Cladosporium fulvum* (syn. *Fulvia fulva*), is a devastating foliar disease particularly prevalent in protected cultivation environments such as greenhouses and plastic sheds (Thomas et al., 1998; Zhao et al., 2022). The pathogen primarily attacks leaves, leading to chlorosis, necrosis, and extensive mold growth on the abaxial leaf surface, which severely impairs photosynthesis and ultimately reduces fruit yield and quality (Thomma et al., 2005; Wang et al., 2018). Under favorable conditions, the disease can cause yield losses of 10-25% annually and, in severe cases, complete crop failure (Mersha et al., 2014). The rapid spread of *C. fulvum* and the emergence of new physiological races that overcome host resistance have made the management of tomato leaf mold a persistent challenge (Rivas and Thomas, 2005).

Conventional control strategies primarily rely on chemical fungicides. However, frequent and excessive application of these chemicals has led to the development of fungicide resistance in pathogen populations, environmental contamination, and potential risks to human health (Sudermann et al., 2022). Cultural practices, including crop rotation, removal of infected plant debris, and the use of resistant cultivars, provide some level of control but are often insufficient due to the rapid evolution of pathogen races and the limited durability of single-gene resistance (Rivas and Thomas, 2005; Zhao et al., 2022). Therefore, there is an urgent need for sustainable and environmentally friendly alternatives, among which biological control has emerged as a promising approach (Kabir et al., 2024; Shi et al., 2024). *Trichoderma* species are among the most extensively studied and commercially applied biocontrol fungi (Dong and Ahammed, 2026; Xue et al., 2025). They are widely distributed in soil and plant rhizospheres and exhibit multiple mechanisms of action against plant pathogens, including mycoparasitism, antibiosis, competition for nutrients and space, and induction of systemic resistance in host plants (Druzhinina et al., 2011; Harman et al., 2004; Poveda, 2021). Several *Trichoderma* strains, such as *T. harzianum*, *T. viride*, and *T. asperellum*, have been shown to suppress various fungal diseases in tomato, including leaf mold, gray mold, and Fusarium wilt (Belabess et al., 2024; Lopez Bucio et al., 2015). For instance, *T. asperellum* has been reported to exhibit strong antagonistic activity against *C. fulvum in vitro*, significantly inhibiting mycelial growth and reducing disease incidence under greenhouse conditions (Belabess et al., 2024). In addition to direct antagonism, *Trichoderma* can colonize plant roots and trigger induced systemic resistance (ISR), which is associated with enhanced activities of defense-related enzymes such as peroxidase (POD), polyphenol oxidase (PPO), and chitinase, as well as the accumulation of phytoalexins and pathogenesis-related proteins (Tyskiewicz et al., 2022; Zhang et al., 2017). Despite these advantages, the efficacy of *Trichoderma* as a standalone biocontrol agent can be influenced by environmental conditions and pathogen pressure, prompting the exploration of integrated approaches that combine beneficial microbes with resistance-inducing compounds.

Melatonin (N-acetyl-5-methoxytryptamine) is a ubiquitous signaling molecule that plays critical roles in plant growth, development, and stress responses (Arnao and Hernandez-Ruiz, 2014; Shi et al., 2016; Sun et al., 2021). Exogenous application of melatonin has been shown to enhance plant tolerance to a wide range of abiotic stresses, including drought, salinity, extreme temperatures, and heavy metal toxicity, primarily by scavenging reactive oxygen species (ROS), increasing antioxidant enzyme activities, and modulating gene expression (Qi et al., 2018; Siddiqui et al., 2019; Wang et al., 2013). More recently, melatonin has also been implicated in plant defense against biotic stresses. For example, melatonin treatment reduced the severity of *Alternaria* black spot in radish and downy mildew in cucumber (Hao et al., 2025; Mandal et al., 2018). The protective effect of melatonin is attributed to its direct antioxidant activity as well as its ability to prime defense responses, including the activation of mitogen-activated protein kinase (MAPK) cascades and the accumulation of phenolic compounds (Cheng et al., 2026; Mansoor et al., 2024; Tiwari et al., 2022). Notably, melatonin biosynthesis in plants involves several key enzymes, including caffeic acid O-methyltransferase (COMT), which catalyzes the final step in the conversion of serotonin to melatonin (Back, 2021; Park et al., 2013). Silencing of the *COMT* gene has been shown to reduce endogenous melatonin levels and compromise stress tolerance, highlighting the importance of this pathway in plant resistance (Ahammed et al., 2018; Park et al., 2013; Zhang et al., 2024). Given the complementary modes of action of *Trichoderma* and melatonin, the former acting as a root-colonizing biocontrol agent that primes systemic defenses and the latter functioning as a direct ROS scavenger and defense elicitor, their combination may offer synergistic effects against foliar pathogens. However, whether *Trichoderma* and melatonin act cooperatively to enhance tomato resistance against *C. fulvum* and whether this enhanced resistance depends on endogenous melatonin synthesis remain largely unknown.

In this study, we screened ten *Trichoderma* strains and identified the *T. asperellum* T141 strain as the most effective antagonist against *C. fulvum*, and determined 100 μmol/L melatonin as the optimal concentration. We then investigated the synergistic effects of combined *T. asperellum* and melatonin application on tomato leaf mold resistance. Our results show that the combined treatment significantly alleviated disease severity, promoted plant growth, reduced oxidative damage, enhanced antioxidant enzyme activities, and improved photosynthetic performance. Furthermore, using virus-induced gene silencing (VIGS) of *COMT1*, a key gene in melatonin biosynthesis, we demonstrated that *COMT1*-dependent melatonin synthesis is essential for *T. asperellum*-induced resistance. Unlike previous studies that broadly implicated melatonin in plant stress responses, our work uniquely demonstrates that *COMT1*-dependent melatonin biosynthesis is mechanistically required for *Trichoderma*-induced resistance to tomato leaf mold, thus advancing understanding of how microbial biocontrol agents and endogenous signaling pathways converge to protect crops. Overall, this study provides a theoretical basis for integrating *T. asperellum* and melatonin as an eco-friendly strategy to control tomato leaf mold.

## 2 Materials and methods

### 2.1 Plant materials and growth conditions

Tomato (*S. lycopersicum* L. cv. ‘Guyu Kaixuan’) seeds were surface-sterilized by soaking in warm water (52 °C) for 20 min, then germinated in a growth chamber at 28 °C. The germinated seeds were sown in 72-well trays filled with sterilized substrate and grown in a controlled environment chamber with a 12 h light/12 h dark photoperiod, day/night temperatures of 27/19 °C, and a relative humidity of 70-80%. At the three-leaf stage, seedlings were transplanted into 10-cm diameter plastic pots (one seedling per pot) and further grown until the four-leaf stage before treatments.

### 2.2 Pathogen and *Trichoderma* cultures

The tomato leaf mold pathogen *Cladosporium fulvum* was originally isolated from infected tomato plants in the Laboratory of Stress Biology of Horticultural Crops at Henan University of Science and Technology, and maintained on potato dextrose agar (PDA) at 4 °C. For inoculation, *C. fulvum* conidia were harvested from 10-day-old PDA cultures, suspended in sterile distilled water, and adjusted to a concentration of 1 × 10^6^ conidia·mL^-1^ using a hemocytometer.

Ten *Trichoderma* strains (T29, TL, T18, T24, T131, T36, T154, T141, T177, and T8) were provided by Zhejiang University. Each strain was activated on PDA at 28 °C for 5 days. For antagonism assays, a 5-mm mycelial plug of *C. fulvum* was placed on one side of a 9-cm PDA plate and incubated at 28 °C for 6 days. Thereafter, a 5-mm plug of each *Trichoderma* strain was placed on the opposite side of the same plate. The plates were further incubated for 7-8 days, and the inhibition rate was calculated based on the radius of the pathogen colony in the treatment relative to the pathogen-alone control. All assays were performed with three replicates.

### 2.3 Melatonin treatment and concentration optimization

Melatonin (MT; purity ≥98%, Sangon Biotech, Shanghai, China) was dissolved in a small volume of ethanol and then diluted with sterile distilled water to final concentrations of 50, 100, 150, 200, and 250 μmol/L. Tomato seedlings at the four-leaf stage were sprayed uniformly on both sides of the leaves with 5 mL per plant of each MT solution one time per day for three consecutive days. Sterile distilled water containing an equal ratio of ethanol, used to prepare various concentrations of melatonin, was used as the control. After MT treatment, the plants were inoculated with *C. fulvum* spore suspension (1 × 10^6^ conidia·mL^-1^, 5 mL per plant). Disease index was assessed 20 days post-inoculation as described below. Among the tested concentrations, 100 μmol/L MT consistently produced the lowest disease index and oxidative damage markers without causing visible phytotoxicity. Therefore, this concentration was selected for subsequent experiments.

### 2.4 Experimental design for combined treatments

Eight treatments were established: Control (sterile water only), CF (*C. fulvum* only), T (*T. asperellum* only), MT (100 μmol/L melatonin only), T + MT, CF + T, CF + MT, and CF + T + MT. For *Trichoderma* treatments, a spore suspension of *T. asperellum* (1 × 10^8^ conidia·mL^-1^) was applied as a root drench (10 mL per plant) through holes made in the rhizosphere substrate 7 days before pathogen inoculation. Melatonin (100 μmol/L) was sprayed uniformly on both leaf surfaces once daily for 3 consecutive days, starting 3 days before pathogen inoculation. Pathogen inoculation with *C. fulvum* spore suspension was performed after the completion of *T. asperellum* and MT applications. Control plants were treated with equal volumes of sterile water at the corresponding time points. Each treatment consisted of 10 plants per replicate, with three independent replicates.

### 2.5 Disease index and control efficacy

Disease severity was evaluated 20 days after pathogen inoculation using a 0–9 scale based on the percentage of leaf area covered by lesions: 0, no lesions; 1, <5%; 3, 6-10%; 5, 11-20%; 7, 21-50%; 9, >51%. The disease index (DI) was calculated as DI = [Σ (disease grade × number of leaves in that grade)/(total leaves × 9)] × 100. Control efficacy (%) = [(DI of control - DI of treatment)/DI of control] × 100.

### 2.6 Growth parameters

Plant height (from the stem base to the apical meristem), stem diameter (measured at the middle of the stem using a digital caliper), and root length were recorded when disease symptoms became evident (20 days after pathogen inoculation). Ten plants per treatment were measured.

### 2.7 Cell death and histochemical staining

Cell death was visualized by trypan blue staining. Tomato leaves of the same position were immersed in a trypan blue solution (125 mL lactic acid, 125 mL water-saturated phenol, 125 mL glycerol, 125 mL sterile water, and 1.25 g trypan blue), boiled for 1-2  min, and then incubated at room temperature overnight. The stained leaves were destained with chloral hydrate solution (250 g chloral hydrate in 200 mL distilled water) until the green color disappeared, and then examined under a stereomicroscope.

Accumulation of superoxide anion (O_2_^·-^) and hydrogen peroxide (H_2_O_2_) was detected by histochemical staining with nitroblue tetrazolium (NBT) and 3,3′-diaminobenzidine (DAB), respectively. Leaves were incubated in NBT solution (0.25 g NBT in 500 mL of 50 mmol·L^-1^ phosphate buffer, pH 7.8) or DAB solution (0.5 g DAB in 500 mL distilled water, pH 5.8) at room temperature overnight. After staining, the leaves were boiled in 80% ethanol until chlorophyll was completely removed, and then observed.

### 2.8 Measurement of ROS, MDA and electrolyte leakage

H_2_O_2_ content and O_2_^·-^ content were measured as previously described (Jiang et al., 2021; Patterson et al., 1984). Malondialdehyde (MDA) content was assayed using the thiobarbituric acid (TBA) method. For electrolyte leakage (EL), leaf discs (0.1 g) were washed, placed in test tubes with 10 mL of deionized water, and incubated at room temperature for 12 h. The initial electrical conductivity (EC_1_) was measured, and the tubes were then boiled for 30 min. After cooling to room temperature, the final electrical conductivity (EC_2_) was recorded. EL (%) = (EC_1_ / EC_2_) × 100 (Jiang et al., 2022). For each measurement, leaf samples from three independent biological replicates (each containing three plants per treatment) were analyzed.

### 2.9 Antioxidant enzyme activity assay

Leaf samples (0.5 g) were homogenized in 5 mL of ice-cold 50 mmol·L^-1^ phosphate buffer (pH 7.8) containing 1 mmol·L^-1^ EDTA and 2% polyvinylpyrrolidone (PVP). The homogenate was centrifuged at 12,000×g for 20 min at 4 °C, and the supernatant was used for enzyme assays. Superoxide dismutase (SOD) activity was measured by the NBT photoreduction method. Peroxidase (POD) activity was determined by monitoring the oxidation of guaiacol at 470 nm. Catalase (CAT) activity was assayed by following the decomposition of H_2_O_2_ at 240 nm. Ascorbate peroxidase (APX) activity was measured by monitoring the decrease in ascorbate at 290 nm. Enzyme activities were assayed using three independent biological replicates, with three plants per replicate.

### 2.10 Photosynthetic parameters and chlorophyll fluorescence

Photosynthetic parameters, including net photosynthetic rate (Pn), intercellular CO_2_ concentration (Ci), stomatal conductance (Gs), and transpiration rate (Tr), were measured on the second fully expanded leaf from the bottom on a sunny day at noon using a portable photosynthesis system (3051D, China). The measurement conditions were: photosynthetically active radiation at 1000 μmol·m^-2^·s^-1^, leaf temperature at 25 °C, and an ambient CO_2_ concentration of approximately 400 μmol·mol^-1^.

Chlorophyll fluorescence parameters were measured using a PSI FluorPen (Photon Systems Instruments, Czech Republic). Leaves were dark-adapted for 30 min before measurement. The maximum quantum efficiency of PSII (Fv/Fm), actual photochemical efficiency of PSII (ΦPSII), photochemical quenching coefficient (qP), and non-photochemical quenching coefficient (NPQ) were recorded. Measurements were performed on ten plants per treatment, with three independent replicates.

### 2.11 Photosynthetic pigment content

Chlorophyll a, chlorophyll b, and carotenoid contents were determined by ethanol extraction. Leaf discs (0.1 g) were immersed in 10 mL of 95% ethanol and kept in the dark for 24 h until the leaf tissue became white. The absorbance of the extract was measured at 665 nm, 649 nm, and 470 nm using a spectrophotometer, and pigment content was calculated according to Lichtenthaler and Wellburn (1983).

### 2.12 Activities of Rubisco and FBPase

The activities of ribulose-1,5-bisphosphate carboxylase/oxygenase (Rubisco) and fructose-1,6-bisphosphatase (FBPase) were determined using an enzyme-linked immunosorbent assay (ELISA) kit (Ruixin Biotech, Quanzhou, China) following the manufacturer’s protocols.

### 2.13 RNA extraction and quantitative real-time PCR

Total RNA was extracted from tomato leaves using the Universal Plant Total RNA Isolation Kit (Vazyme, Nanjing, China). First-strand cDNA was synthesized using the PrimeScript™ RT Reagent Kit with gDNA Eraser (TaKaRa, Japan). Quantitative real-time PCR was performed with Green qPCR SuperMix (TransGen Biotech, Beijing, China) on a CFX96 Real-Time System (Bio-Rad, USA). The primers used for amplifying the photosynthesis-related genes *FBPase*, *FBP4*, *SBPase*, and *TPI* are listed in **Supplementary Table 1**. The tomato actin gene was used as an internal control. Relative expression levels were calculated using the 2^-ΔΔCt^ method (Livak and Schmittgen, 2001).

### 2.14 Virus-induced gene silencing of *COMT1*

To silence the *COMT1* gene (Solyc03g080180.2), a 298 bp fragment was amplified from tomato cDNA and inserted into the pTRV2 vector using EcoRI and XhoI restriction sites. The recombinant pTRV2-*COMT1* and the helper plasmid pTRV1 were transformed separately into *Agrobacterium tumefaciens* strain GV3101. For VIGS, *Agrobacterium* cultures carrying pTRV1 and pTRV2-*COMT1* (or empty pTRV2 as a control) were mixed at a 1:1 ratio and infiltrated into the cotyledons of two-week-old tomato seedlings using a needleless syringe. The pTRV2-*PDS* construct was used as a visual marker for silencing efficiency (photobleaching phenotype). After the *PDS* control showed white leaves (approximately 15 days post-infiltration), the true leaves of the remaining plants were collected for validation of *COMT1* silencing by qRT-PCR and for determination of endogenous melatonin content using an ELISA kit (Ruixin Biotech, Quanzhou, China). The silenced plants were then subjected to the experimental treatments.

### 2.15 Statistical analysis

All experiments were conducted with three independent biological replicates. Data were expressed as mean ± standard deviation (SD). Statistical analyses were performed using SPSS 21.0 software. Differences among treatments were assessed by one-way analysis of variance (ANOVA) followed by Duncan’s multiple range test at *P* < 0.05.

## 3 Results

### 3.1 Screening of *Trichoderma* strains antagonistic to *Cladosporium fulvum*

**Figure 1:**
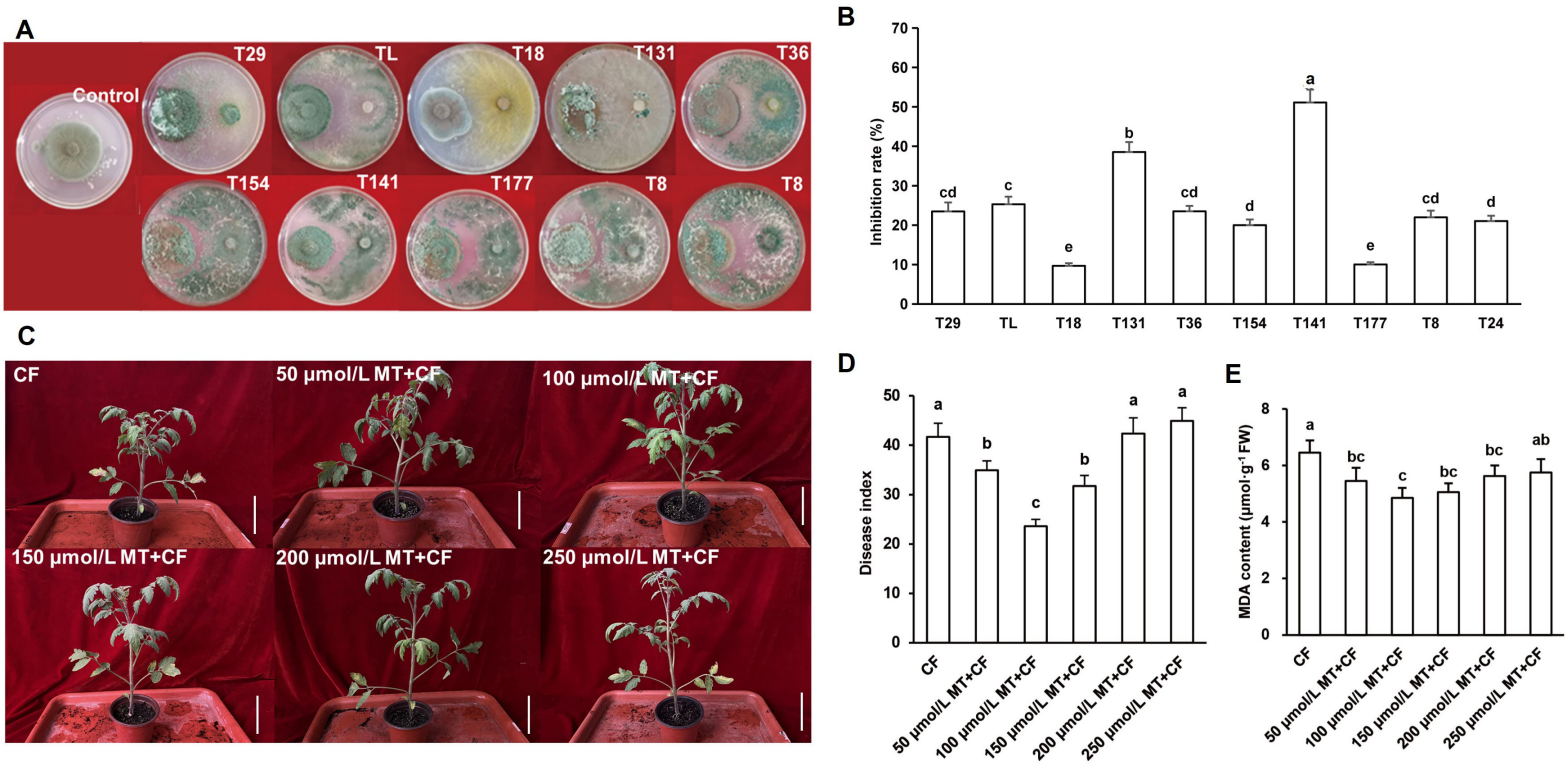
Selection of *Trichoderma* strains and melatonin concentration against *C. fulvum*. **(A)** *In vitro* dual culture assay of ten *Trichoderma* strains against *C. fulvum* on PDA plates. **(B)** Inhibition rates of ten *Trichoderma* strains against *C. fulvum*. Data are means ± SD (n = 8). **(C)** Phenotype of tomato plants inoculated with *C. fulvum* after spraying melatonin at different concentrations. Bar, 9 cm. **(D)** The disease index of tomato plants inoculated with *C. fulvum* after spraying melatonin at different concentrations. **(E)** MDA content in tomato leaves inoculated with *C. fulvum* after spraying melatonin at different concentrations. Data are means ± SD (n = 3). Different letters above bars indicate significant differences according to Duncan’s multiple range test (*P* < 0.05).

Previous studies have demonstrated that *Trichoderma* exhibits strong antagonistic activity against a broad range of fungal pathogens, including *C. fulvum*, the causal agent of tomato leaf mold (Dong and Ahammed, 2026; Harman et al., 2004; Poveda, 2021). To identify a potent biocontrol agent against tomato leaf mold caused by *C. fulvum*, ten *Trichoderma* strains (T29, TL, T18, T24, T131, T36, T154, T141, T177, and T8) were evaluated using a dual culture assay on PDA plates (**Figure 1A**). All strains exhibited varying degrees of inhibitory activity against *C. fulvum* mycelial growth, with inhibition rates ranging from 9.67% to 51.10%. The T18 and T177 strains showed the weakest antagonistic effects, with inhibition rates of 9.67% and 10.03%, respectively, which were not significantly different from each other but were significantly lower than those of most other strains. In contrast, the T141 strain exhibited the strongest inhibitory effect, with an inhibition rate of 51.10%, significantly higher than those of all other tested strains, suggesting strong competitive and possibly mycoparasitic abilities (**Figure 1B**). Therefore, *T. asperellum* T141 strain was used as the biocontrol strain for the subsequent studies.

### 3.2 Effects of melatonin on tomato leaf mold resistance and optimization of exogenous melatonin concentration

Exogenous melatonin has been shown to enhance plant resistance to fungal diseases (Cheng et al., 2026; Mandal et al., 2018). To investigate whether an optimal concentration of melatonin can enhance tomato leaf mold resistance, six concentrations (50, 100, 150, 200, and 250 μmol/L) of melatonin were sprayed onto tomato seedlings, followed by inoculation with a *C. fulvum* spore suspension. Compared to the CF treatment, application of melatonin at varying concentrations alleviated the damage caused by *C. fulvum* infection in tomato plants to varying degrees (**Figure 1C**). At 20 days after inoculation with *C. fulvum*, the disease index of tomato plants was 41.67. Exogenous melatonin at 50-150 μmol/L significantly reduced the disease index of tomato leaf mold. The most significant effects were observed at a melatonin concentration of 100 μmol/L, with a disease index of only 23.61, significantly lower than that in the CF treatment and all other concentration treatments (**Figure 1D**). However, tomato seedlings treated with 200 or 250 μmol/L melatonin showed disease indices of 42.33 and 44.92, respectively, which were not significantly different from the CF treatment, indicating that high concentrations of melatonin failed to confer protection and even slightly aggravated disease severity (**Figure 1D**). Meanwhile, biochemical analysis showed that tomato leaves treated with exogenous 100 μmol/L melatonin had the lowest malondialdehyde (MDA) level. Compared with the CF treatment, MDA content was reduced by 24.85% under 100 μmol/L melatonin. Exogenous application of 50 and 150 μmol/L melatonin also reduced the levels of MDA produced in tomato leaves, while melatonin concentrations exceeding 200 μmol/L failed to reduce this oxidative stress marker (**Figure 1E**). Taken together, exogenous melatonin at 100 μmol/L optimally enhances tomato leaf mold resistance by alleviating oxidative damage. Therefore, 100 μmol/L melatonin was selected for all subsequent experiments.

### 3.3 Synergistic effect of *T. asperellum* and melatonin on induced resistance against tomato leaf mold

**Figure 2:**
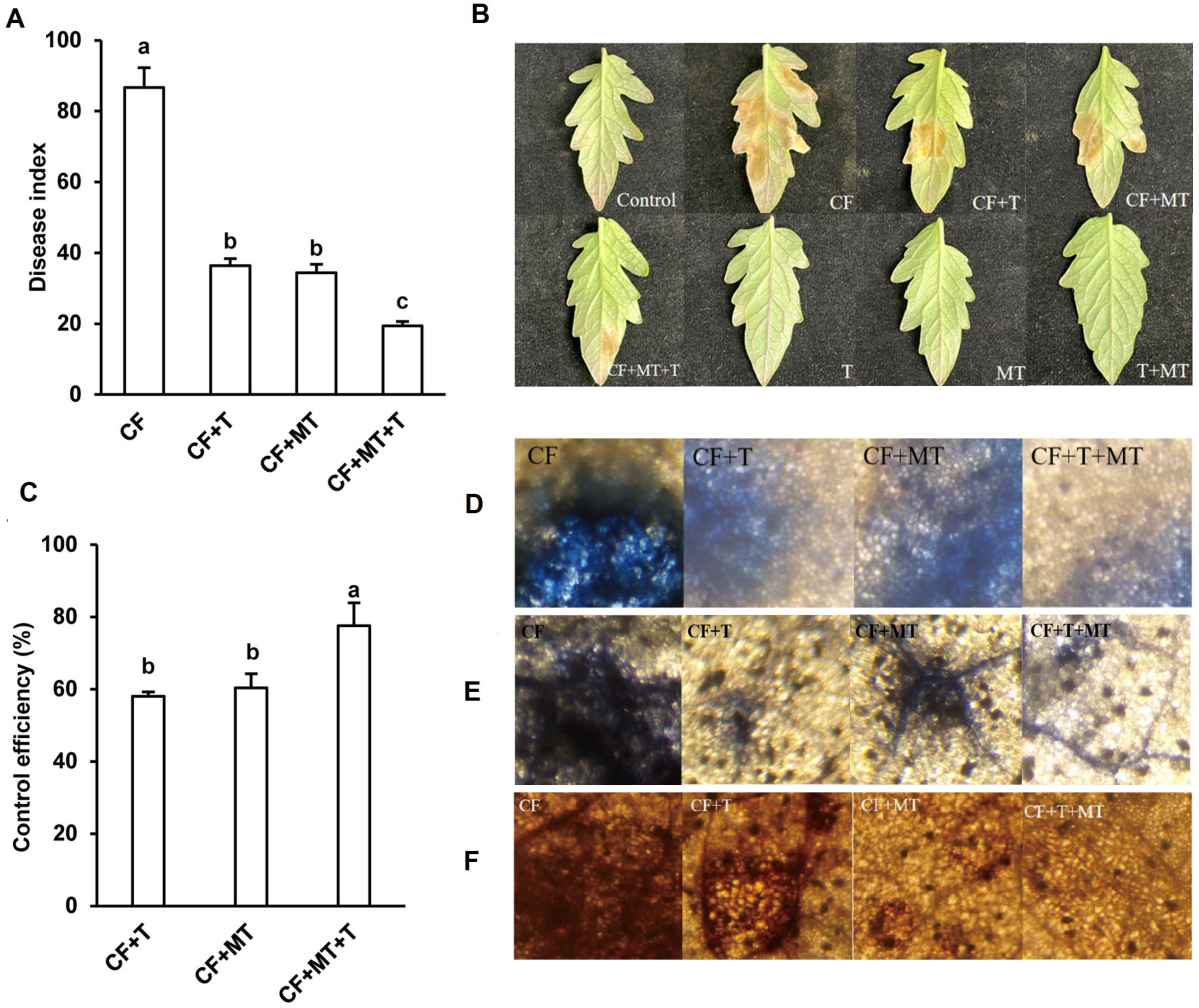
Effects of *Trichoderma asperellum* strain T141 and melatonin on tomato leaf mold resistance. **(A)** Disease index of leaf mold upon treatment with *T. asperellum* and melatonin in tomato plants under CF (*C. fulvum* only), CF+T (treated with *T. asperellum* T141 strain at 7 days before *C. fulvum* inoculation), CF+MT (melatonin pretreatment for 3 consecutive days before *C. fulvum* inoculation), and CF+T+MT (combined treatment with *T. asperellum* T141 strain and melatonin before *C. fulvum* inoculation). Bar, 9 cm. **(B)** Leaf phenotype under various treatments. **(C)** Control efficiency of *T. asperellum* and/or melatonin on tomato leaf mold. **(D)** Trypan blue staining, **(E)** NBT staining, **(F)** DAB staining. Data are means ± SD (n = 3). Different letters above bars indicate significant differences according to Duncan’s multiple range test (*P* < 0.05).

At 20 days post-inoculation, tomato plants in the CF treatment exhibited severe disease symptoms (**Figures 2A, B**). In contrast, tomato plants in the CF+T and CF+MT treatments showed markedly less disease symptoms, indicating that either *T. asperellum* or melatonin alone partially alleviated pathogen-induced damage. Notably, the CF+T+MT treatment displayed the best overall plant growth, suggesting a synergistic protective effect. The plant height, stem diameter, and root length of tomato seedlings were partially restored under CF+T and CF+MT treatments. Crucially, the tomato plants under CF+T+MT treatment showed greater recovery of plant growth parameters relative to CF, with increases of 45.65% in height, 13.44% in stem diameter, and 39.93% in root length, indicating that the combined treatment most effectively alleviated pathogen-induced growth inhibition (**Supplementary Figure 1**).

**Figure 3:**
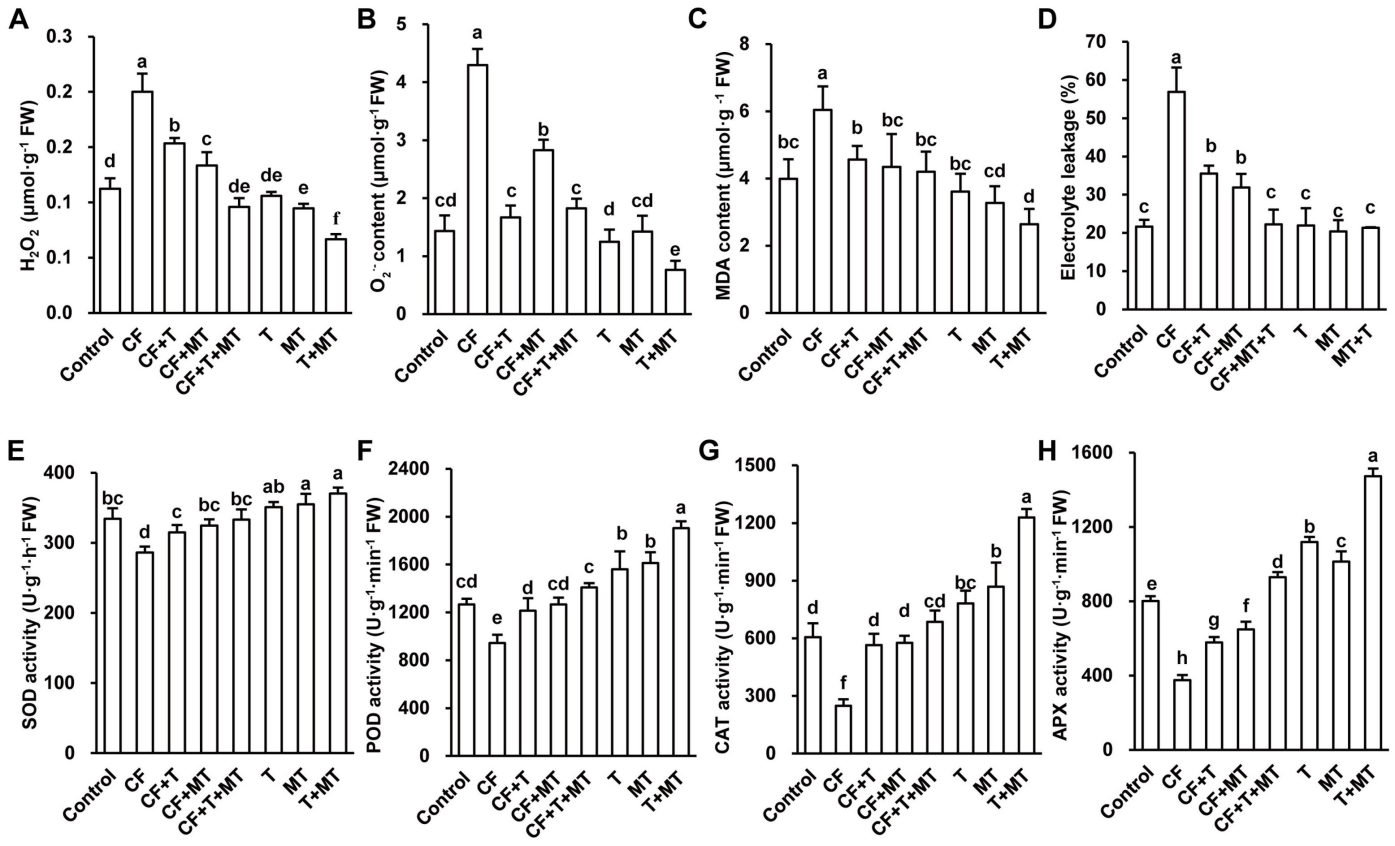
*Trichoderma asperellum* and melatonin alleviate *C. fulvum*-induced oxidative stress in tomato. **(A)** H_2_O_2_ content, **(B)** O_2_^·-^ content, **(C)** MDA content, **(D)** electrolyte leakage, **(E)** SOD activity, **(F)** POD activity, **(G)** CAT activity, and **(H)** APX activity in tomato leaves under Control, CF, CF+T, CF+MT, CF+T+MT, T, MT, and T+MT treatments. Data are means ± SD (n = 3). Different letters above bars indicate significant differences according to Duncan’s multiple range test (*P* < 0.05).

The disease index of tomato plants in the CF treatment was 86.67, whereas it decreased to 34.39 and 36.41 in the CF+T and CF+MT treatments, respectively, with control efficacies of 60.32% and 57.99%, respectively. The combined treatment CF+T+MT further lowered the disease index to 19.44, achieving a control efficacy of 77.57%, significantly higher than either single treatment (**Figures 2A, C**). Trypan blue staining revealed extensive cell death of tomato leaves in CF treatment, which was substantially reduced in CF+T and CF+MT treatments and was minimal in CF+T+MT treatment (**Figure 2D**). NBT and DAB staining showed that CF induced massive accumulation of superoxide anion (O_2_^·-^) and hydrogen peroxide (H_2_O_2_); the levels of both O_2_^·-^ and H_2_O_2_ were markedly reduced in the CF+T and CF+MT treatments, whereas the reactive oxygen species (ROS) accumulation in tomato leaves was further significantly attenuated in the CF+T+MT treatment (**Figures 2E, F**). Quantitatively, H_2_O_2_ and O_2_^·-^ contents in tomato leaves under the CF+T+MT treatment decreased by 52% and 57.55%, respectively, compared with the CF treatment (**Figures 3A, B**). Membrane integrity was also better preserved by the combined treatment. *C. fulvum* caused an increase of 51.41% in MDA levels and a 162.57% rise in electrolyte leakage in tomato leaves. The application of *T. asperellum* or melatonin significantly reduced MDA content and electrolyte leakage in tomato leaves, while the combined application of both agents further decreased electrolyte leakage (**Figures 3C, D**). Application of *T. asperellum* and melatonin significantly increased the activities of antioxidant enzymes in tomato leaves under pathogen stress. Compared with the CF treatment, the combined treatment (CF+T+MT) significantly elevated SOD, POD, CAT, and APX activities by 16.35%, 49.21%, 176.34%, and 148%, respectively (**Figures 3E–H**). Collectively, these results demonstrate that the synergistic application of *T. asperellum* and melatonin not only improves plant growth and reduces disease severity but also enhances antioxidant enzyme activities, thereby conferring greater resistance against tomato leaf mold.

### 3.4 Synergistic effects of *Trichoderma* and melatonin treatment on photosynthesis in *C. fulvum*-inoculated tomato plants

**Figure 4:**
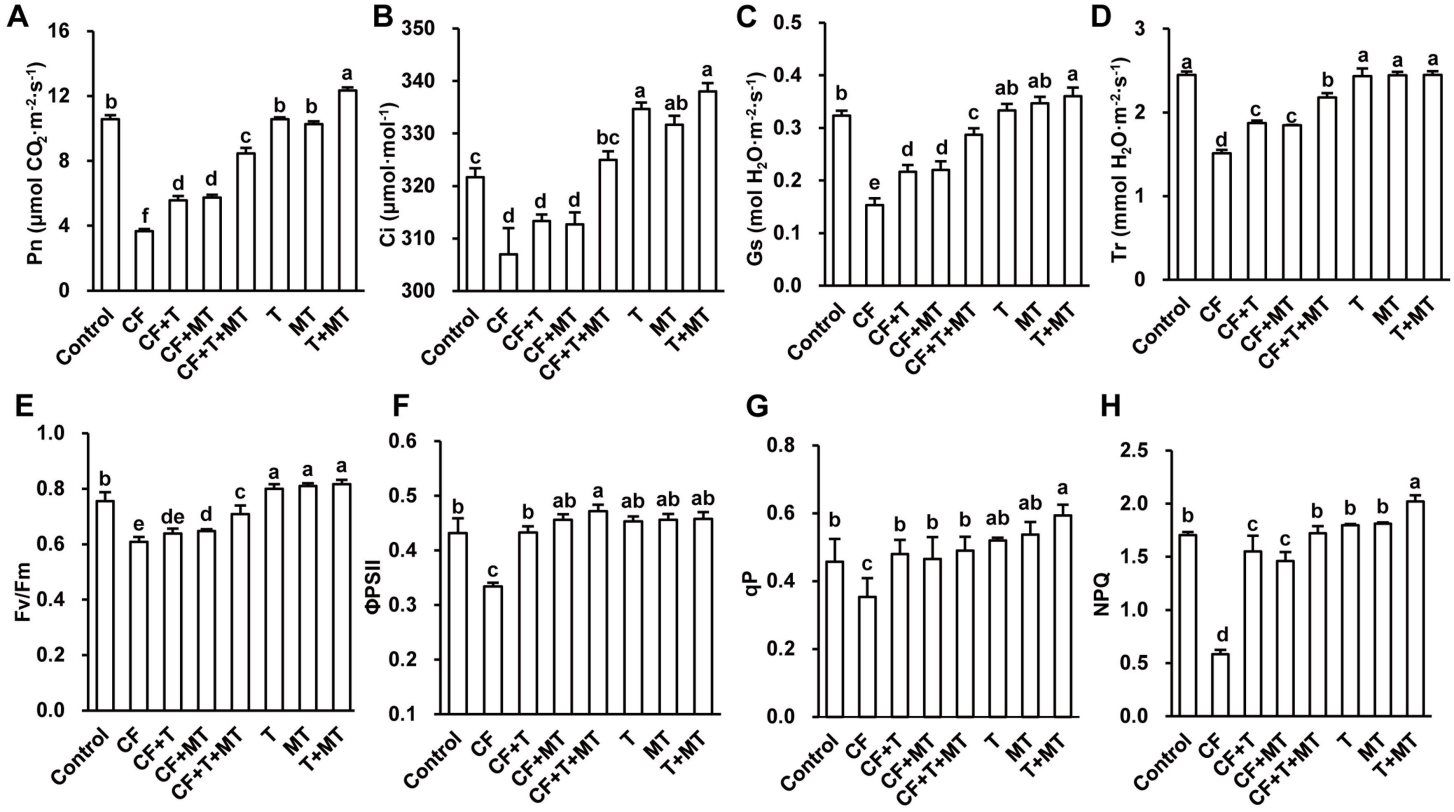
Leaf gas exchange and chlorophyll fluorescence parameters of tomato leaves inoculated with *C. fulvum* under combined treatment with *T. asperellum* and melatonin. **(A)** net photosynthetic rate (Pn), **(B)** Intercellular CO_2_ concentration (Ci), **(C)** stomatal conductance (Gs), **(D)** transpiration rate (Tr), **(E)** the maximum photochemical efficiency of PSII in dark-adapted leaves (Fv/Fm), **(F)** the effective quantum yield under light-adapted conditions (ΦPSII), **(G)** photochemical quenching coefficient (qP), and **(H)** non-photochemical quenching coefficient (NPQ) in tomato leaves under Control, CF, CF+T, CF+MT, CF+T+MT, T, MT and T+MT treatments. Data are means ± SD (n = 3). Different letters above bars indicate significant differences according to Duncan’s multiple range test (*P* < 0.05).

**Figure 5:**
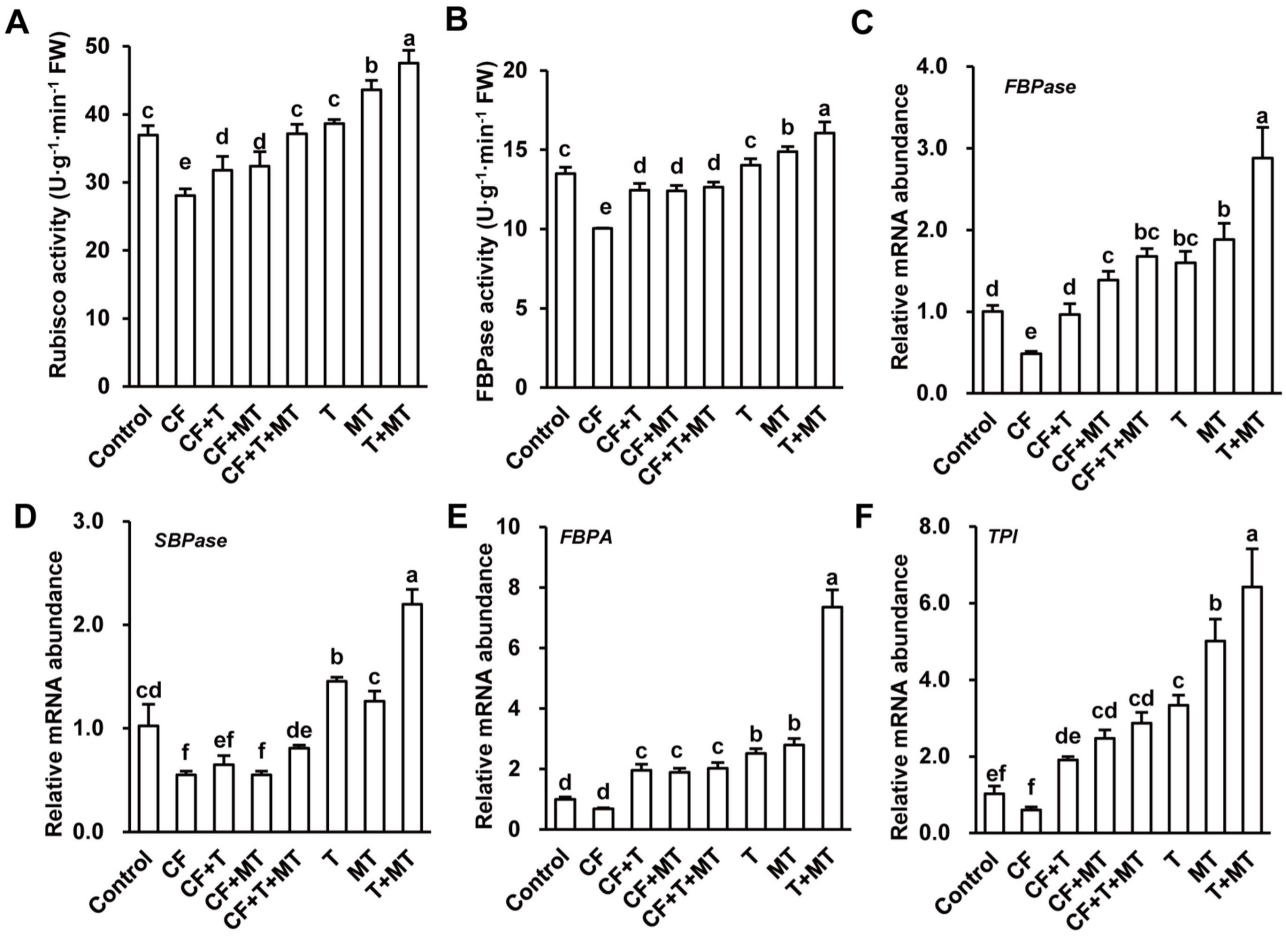
Photosynthetic enzyme activity and gene expression in tomato leaves as influenced by *T. asperellum* and/or melatonin treatment under *C. fulvum* stress. **(A)** Rubisco activity, **(B)** FBPase activity, Relative mRNA abundance of **(C)** *FBPase*, **(D)** *SBPase*, **(E)** *FBPA*, and **(F)** *TPI* in tomato leaves under Control, CF, CF+T, CF+MT, CF+T+MT, T, MT and T+MT treatment. Data are means ± SD (n = 3). Different letters above bars indicate significant differences according to Duncan’s multiple range test (*P* < 0.05).

Since tomato leaf mold primarily attacks plant leaves and is known to impair photosynthetic performance (Dene et al., 2023; Wang et al., 2018), we examined the effects of pre-application of *T. asperellum* and melatonin on key photosynthetic parameters. Compared with the Control, *C. fulvum* infection significantly reduced photosynthetic pigment contents, including chlorophyll a, chlorophyll b, and carotenoids (**Supplementary Figure 2**). In contrast, individual application of *T. asperellum*, melatonin, or their combination notably increased these pigment levels relative to the control, with the T+MT treatment showing the highest values (**Supplementary Figure 2**). Similarly, *C. fulvum* treatment markedly decreased Pn, Ci, Gs, and Tr, as well as chlorophyll fluorescence parameters including Fv/Fm, ΦPSII, qP, and NPQ, compared with the control (**Figures 4A–H**). By contrast, T, MT, and T+MT treatments significantly enhanced these photosynthetic parameters in healthy plants, with the combined treatment (T+MT) exhibiting the strongest stimulatory effect (**Figures 4A–H**). Pre-application of *T. asperellum* or melatonin significantly increased photosynthetic pigment contents compared with CF alone. CF+T and CF+MT elevated chlorophyll a, chlorophyll b, and carotenoid levels. The combined treatment CF+T+MT resulted in the greatest enhancement, with chlorophyll a increased by 133.33%, chlorophyll b by 142.30%, and carotenoids by 43.81% relative to CF (**Supplementary Figure 2**). Compared with CF, CF+T increased Pn, Ci, Gs, and Tr by 51.82%, 4.04%, 43.48%, and 24.06%, respectively. CF+MT increased these parameters by 56.36%, 3.11%, 22.30%, and 41.30%, respectively. The combined treatment CF+T+MT produced the strongest effects, with Pn, Ci, Gs, and Tr increasing by 130.91%, 5.86%, 86.96%, and 44.37%, respectively, relative to CF (**Figures 4A–D**). The treatments also enhanced chlorophyll fluorescence parameters. CF+T and CF+MT partially restored Fv/Fm, ΦPSII, qP, and NPQ. CF+T+MT further increased these parameters by 16.31%, 41.08%, 38.68%, and 83.33%, respectively, compared with CF (**Figures 4E–H**). The activities of Calvin-cycle enzymes Rubisco and FBPase were markedly lower in CF plants than in the control, while T, MT, and T+MT treatments increased their activities. Both CF+T and CF+MT significantly elevated Rubisco and FBPase activities, and CF+T+MT increased Rubisco and FBPase activities by 32.33% and 25.91%, respectively, relative to CF (**Figures 5A, B**). Meanwhile, the expression levels of four photosynthesis-related genes (*FBPase*, *SBPase*, *FBPA*, and *TPI*) were lower in CF plants than in the control. In contrast, T, MT, and T+MT treatments upregulated their expression. CF+T and CF+MT partially restored their expression, whereas CF+T+MT strongly upregulated these genes by 245.83%, 46.93%, 196.70%, and 373.15%, respectively, compared with CF (**Figures 5C–F**). Collectively, these results demonstrate that pre-application of *T. asperellum* and melatonin, particularly in combination, effectively alleviates *C. fulvum*-induced photosynthetic impairment by preserving photosynthetic pigments, enhancing gas exchange, protecting PSII integrity, increasing Calvin-cycle enzyme activities, and upregulating key photosynthetic genes.

### 3.5 Involvement of the *COMT1* gene in *T. asperellum*-induced resistance against tomato leaf mold

**Figure 6:**
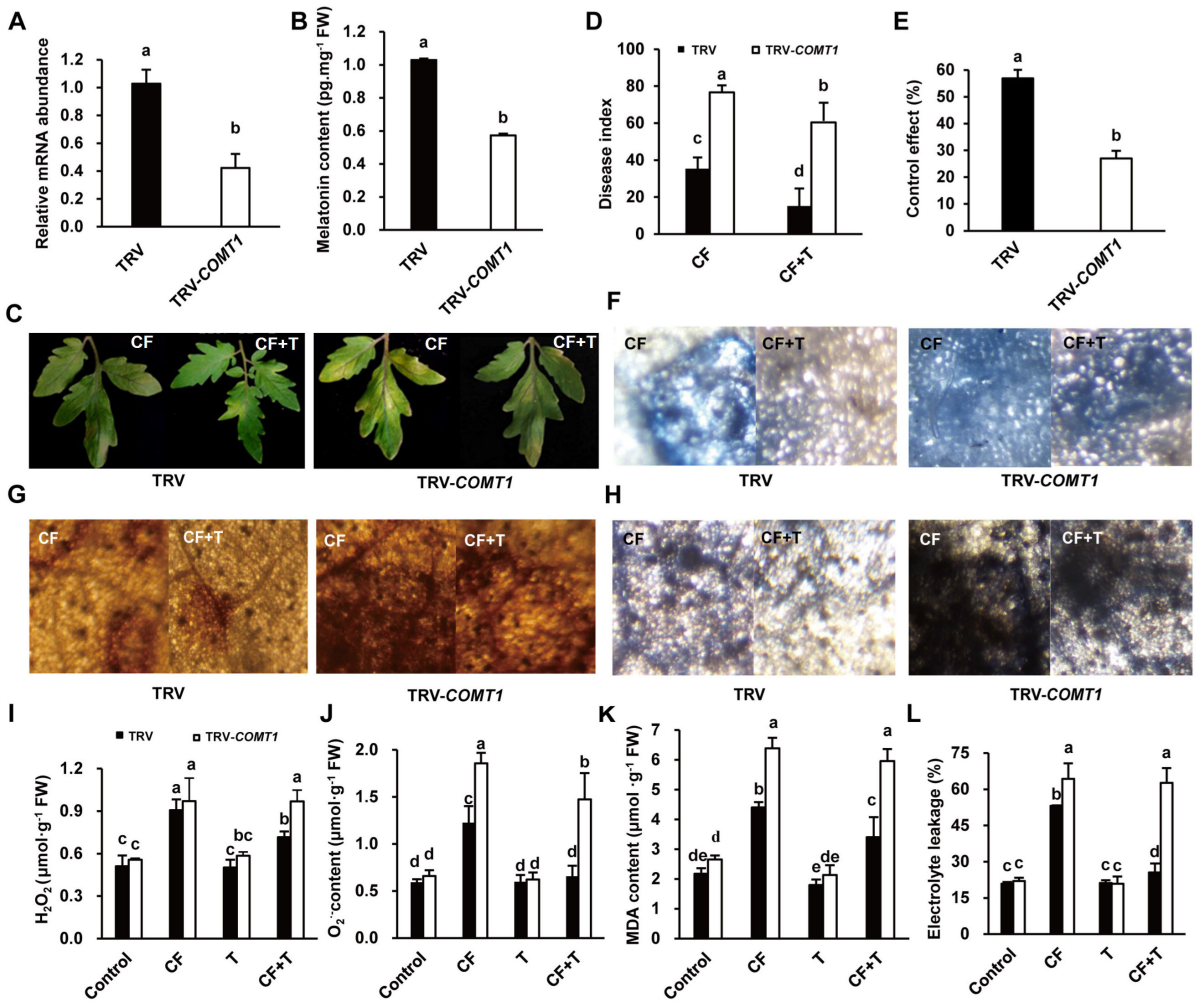
*COMT1* gene silencing reduces melatonin accumulation, aggravates oxidative stress and compromises *T. asperellum-*induced disease resistance to *C. fulvum*. **(A)** The expression level of *COMT1* gene in TRV and TRV-*COMT1* plants. **(B)** Melatonin content in TRV and TRV-*COMT1* plants. **(C)** Leaf phenotype of TRV and TRV-*COMT1* tomato plants under CF and CF+T. **(D)** Disease index of TRV and TRV-*COMT1* tomato plants under CF and CF+T. **(E)** Control efficiency of *T. asperellum* against tomato leaf mold in TRV and TRV-*COMT1* tomato plants. **(F)** Trypan blue staining, **(G)** DAB staining, **(H)** NBT staining, **(I)** H_2_O_2_ content, **(J)** O_2_^·-^ content, **(K)** MDA content, and **(L)** electrolyte leakage in leaves of TRV and TRV-*COMT1* tomato plants. Data are means ± SD (n = 3). Different letters above bars indicate significant differences according to Duncan’s multiple range test (*P* < 0.05).

To determine whether the melatonin biosynthesis pathway is required for *T. asperellum*-induced resistance, we silenced the *COMT1* gene using VIGS. Compared with the empty vector control (TRV), *COMT1*-silenced plants (TRV-*COMT1*) showed a significant reduction in *COMT1* transcript levels and a marked decrease in endogenous melatonin content (**Figures 6A, B**). Under *C. fulvum* stress, TRV-*COMT1* plants had significantly reduced plant height and stem diameter compared to TRV plants. *T. asperellum* treatment improved growth in TRV plants but failed to rescue growth in TRV-*COMT1* plants (**Supplementary Figure 3**).

**Figure 7:**
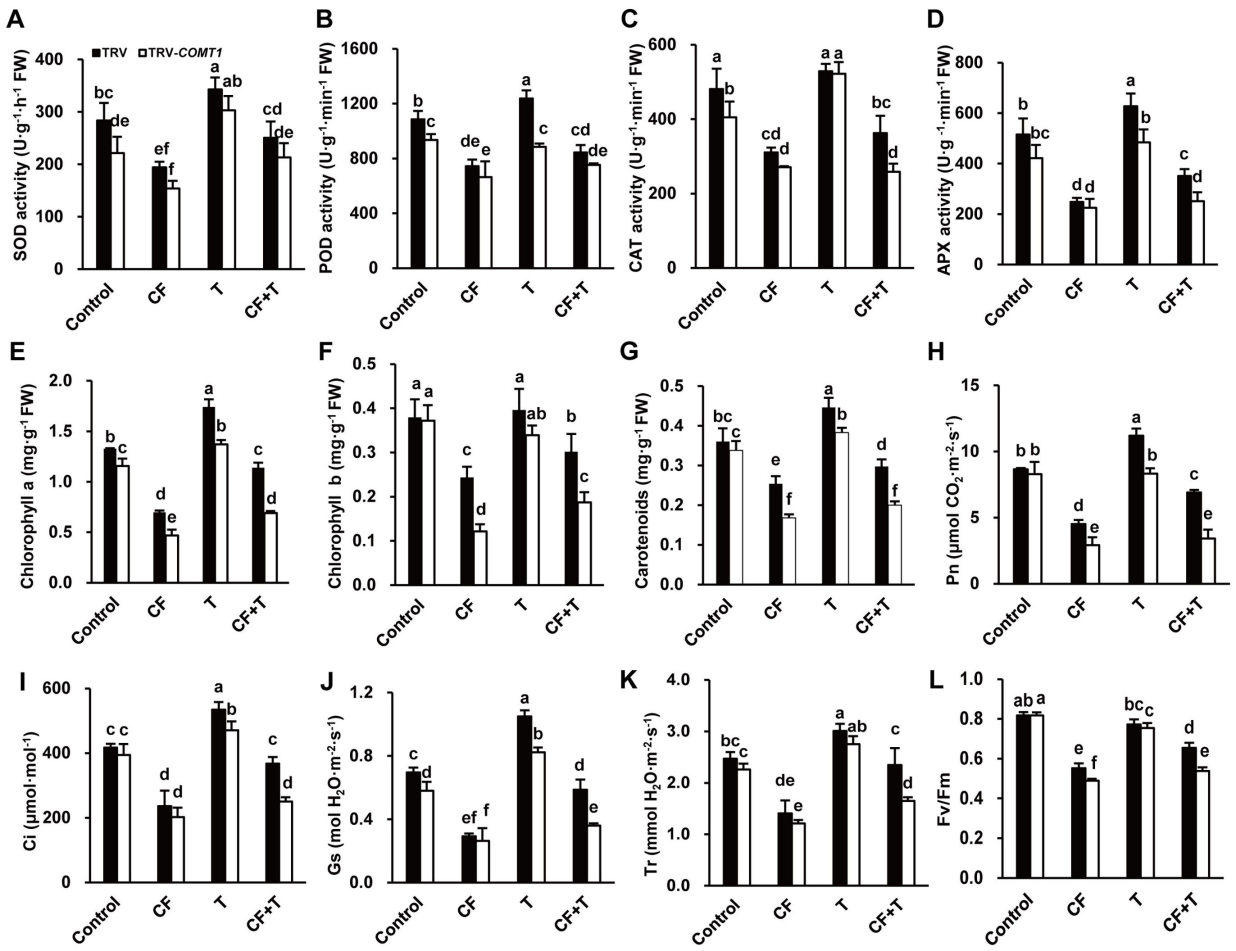
*COMT1* gene silencing compromises *T. asperellum*-induced antioxidative defense and photosynthetic recovery under *C. fulvum* infection in tomato leaves. **(A)** SOD activity, **(B)** POD activity, **(C)** CAT activity, **(D)** APX activity, **(E)** chlorophyll a content, **(F)** chlorophyll b content, **(G)** carotenoids content, **(H)** Pn, **(I)** Ci, **(J)** Gs, **(K)** Tr, and **(L)** Fv/Fm in TRV and TRV-*COMT1* tomato plants under Control, CF, T and CF+T. Data are means ± SD (n = 3). Different letters above bars indicate significant differences according to Duncan’s multiple range test (*P* < 0.05).

At 20 days post-inoculation, TRV plants exhibited severe disease symptoms in the CF treatment, while TRV-*COMT1* plants showed even more pronounced yellowing and mold in the CF treatment. Treatment with *T. asperellum* (CF+T) significantly alleviated symptoms in TRV plants but had limited effect in *COMT1*-silenced plants (**Figure 6C**). TRV plants had a disease index of 35.31, whereas TRV-*COMT1* plants had a much higher index of 76.67. With *T. asperellum* treatment, the disease index was reduced to 15.24 in TRV plants, corresponding to a control efficacy of 56.83%. In contrast, application of *T. asperellum* reduced the disease index of TRV-*COMT1* plants to 60.37, with a control efficacy of only 27.0% (**Figures 6D, E**). Cell death was extensive in TRV-*COMT1* leaves and only marginally reduced by *T. asperellum* treatment, whereas TRV leaves showed much less cell death (**Figure 6F**). NBT and DAB staining revealed that TRV-*COMT1* leaves accumulated massive amounts of O_2_^·-^ and H_2_O_2_. *T. asperellum* only partially alleviated this accumulation in the *COMT1*-silenced tomato plants (**Figures 6G, H**). Quantitative measurements showed that TRV-*COMT1* plants had significantly higher O_2_^·-^ contents than TRV plants under *C. fulvum* stress. Application of *T. asperellum* reduced H_2_O_2_ in TRV plants but not in TRV-*COMT1* plants (**Figure 6I**). TRV-*COMT1* plants also exhibited significantly higher MDA content and electrolyte leakage than TRV plants. *T. asperellum* treatment significantly reduced MDA and electrolyte leakage in TRV plants, but failed to reduce these membrane damage markers in TRV-*COMT1* plants (**Figures 6K, L**). Compared with TRV plants, TRV-*COMT1* plants showed lower antioxidant enzyme activities, including SOD, POD, CAT, and APX. *T. asperellum* treatment significantly increased SOD, CAT, and APX activities in TRV plants, but the induction in CAT and APX activities, was compromised in TRV-*COMT1* plants (**Figures 7A–D**).

Photosynthetic parameters were also affected by silencing of the *COMT1* gene. TRV-*COMT1* plants had lower chlorophyll a, chlorophyll b, and carotenoid contents compared with TRV plants under pathogen stress. *T. asperellum* treatment partially restored pigment levels in TRV plants but had a limited effect in TRV-*COMT1* plants (**Figures 7E–G**). Moreover, TRV-*COMT1* plants had significantly lower Pn than TRV plants under pathogen stress. *T. asperellum* treatment significantly improved Pn, Gs, and Tr in TRV plants, but this recovery was largely lost in TRV-*COMT1* plants. Similarly, Fv/Fm was lower in TRV-*COMT1* plants under *C. fulvum* stress and was just partly restored by *T. asperellum* in the *COMT1*-silenced plants (**Figures 7H–L**). Overall, these results demonstrate that *COMT1*-dependent melatonin synthesis is indispensable for *T. asperellum*-induced resistance against tomato leaf mold, affecting disease severity, oxidative stress, membrane integrity, antioxidant defense, and photosynthetic performance.

## 4 Discussion

The present study demonstrates that the combination of *T. asperellum* and exogenous melatonin exerts a strong synergistic effect against tomato leaf mold. Compared with pathogen-only controls (CF), the combined treatment (CF+T+MT) substantially reduced disease severity and markedly improved plant growth (**Figures 2A–C**; **Supplementary Figure 1**). Single treatments were less effective than the combination, indicating that the combination significantly outperforms individual applications. This synergy likely arises from complementary modes of action. *Trichoderma* colonizes roots and primes systemic defense mechanisms (Gulzar et al., 2023; Jaiswal et al., 2020; Lopez Bucio et al., 2015), while melatonin acts as a direct ROS scavenger and local defense elicitor on leaves (Asad, 2022; La Spada et al., 2020; Zeng et al., 2022). Similar synergistic effects have been reported for *T. harzianum* combined with melatonin against salt toxicity in watermelon (Ghani et al., 2024). Because melatonin was applied foliarly and *Trichoderma* as a root drench, direct interaction between the two agents was minimized in our study. The observed synergy therefore reflects plant-mediated responses rather than altered microbial growth. Our data suggest that the dual-layer strategy, root-based priming and foliar-based direct protection, is particularly effective for foliar pathogens such as *C. fulvum*, which directly invade photosynthetic tissues.

A central finding of this work is that *COMT1*-dependent melatonin biosynthesis is indispensable for *T. asperellum*-induced resistance. Virus-induced gene silencing of the *COMT1* gene drastically reduced melatonin content (**Figure 6B**) and dramatically increased susceptibility to *C. fulvum*. Silencing of *COMT1* dramatically increased disease susceptibility (**Figure 6D**). More importantly, the protective effect of *T. asperellum* was largely compromised in *COMT1*-silenced plants, with control efficacy markedly reduced compared to TRV plants (**Figure 6E**). This is the first direct genetic evidence that a biocontrol fungus requires host melatonin to mount a full defense against a foliar pathogen. Consistently, *COMT1* silencing also impaired the ability of *T. asperellum* to reduce MDA levels and electrolyte leakage (**Figures 6K, L**) and to restore antioxidant enzyme activities (**Figures 7A–D**). These results position melatonin as a critical downstream signal in *T. asperellum*-triggered resistance. Studies in other systems have shown that *COMT1*-deficient plants are more susceptible to bacterial and fungal pathogens, and that beneficial microbes such as arbuscular mycorrhizal fungi depend on host jasmonate pathways (Li et al., 2025; Shan et al., 2025). Our findings extend this concept by identifying melatonin as an essential host factor for *T. asperellum* efficacy.

Pathogen attack typically triggers a rapid burst of ROS, causing membrane lipid peroxidation, electrolyte leakage, and programmed cell death (Mittler et al., 2022). In our study, the CF treatment led to elevated levels of H_2_O_2_ and O_2_^·-^, accompanied by increased MDA content and electrolyte leakage (**Figure 3**). Pre-application of *T. asperellum* or melatonin significantly reduced these oxidative markers, but the combined treatment (CF+T+MT) was most effective. The reduction in oxidative damage correlated strongly with enhanced activities of SOD, POD, CAT, and APX (**Figure 3**). Notably, the magnitude of enzyme induction in the combined treatment exceeded additive effects, indicating true synergy. This pattern is consistent with independent reports that *T. harzianum* boosts antioxidant capacity in tomato against *Botrytis cinerea* (Geng et al., 2022) and that melatonin enhances ascorbate-glutathione cycle enzymes in wheat under salt stress (Talaat and Todorova, 2022). Our results further show that *COMT1* silencing largely eliminated the antioxidant enzyme induction by *T. asperellum* (**Figure 7**), confirming that endogenous melatonin is required for this effect. Mechanistically, *T. asperellum* and melatonin may converge on activating transcription factors such as WRKY and NAC, which regulate a battery of antioxidant genes (Nuruzzaman et al., 2013; Wani et al., 2021). In addition, melatonin can directly neutralize multiple ROS species (Arnao and Hernandez-Ruiz, 2021), providing a rapid first-line defense. The dual action of direct scavenging plus transcriptional upregulation explains the superior oxidative stress mitigation observed in tomato plants under the CF+T+MT treatment.

Photosynthesis is highly sensitive to biotic stress, and preserving photosynthetic capacity is essential for plant survival and yield (Brestic and Allakhverdiev, 2022). In our study, *C. fulvum* infection severely reduced leaf gas exchange (Pn, Gs, Tr) and chlorophyll fluorescence parameters (Fv/Fm, ΦPSII, qP, and NPQ), and markedly decreased chlorophyll a, chlorophyll b, and carotenoid contents (**Figure 4**; **Supplementary Figure 2**). The combined treatment (CF+T+MT) not only restored these parameters but also increased the activities of Calvin-cycle enzymes Rubisco and FBPase and strongly upregulated the expression of photosynthesis-related genes *FBPase*, *SBPase*, *FBPA*, and *TPI* (**Figure 5**). This multi-level recovery from pigment stabilization to gene expression explains why tomato plants under the CF+T+MT treatment grew much better than those under the other inoculated treatments. The improvement in Pn and Gs likely reflects enhanced stomatal function, which may be attributed to *Trichoderma*-mediated improvement in root hydraulic conductivity and hormonal balance, particularly abscisic acid signaling (Basharat et al., 2025; Wu et al., 2022; Zhou et al., 2016). Meanwhile, the recovery of Fv/Fm and ΦPSII suggests that the combined treatment protects PSII reaction centers from photo-oxidative damage. Melatonin is known to stabilize photosynthetic complexes and scavenge ROS generated in the electron transport chain, thereby preserving chloroplast integrity (Ahammed et al., 2024). The upregulation of photosynthesis-related genes further indicates that transcriptional reprogramming contributes to the observed recovery. The recovery of photosynthetic parameters in combined treatments reflects melatonin’s antioxidant role and *Trichoderma*’s ISR effect, which together protect PSII integrity and sustain Calvin cycle enzyme activities. Similar photosynthetic rescue by *T. asperellum* has been observed in tomato infected by *Cucumber mosaic virus* (Vitti et al., 2016) and by melatonin in maize under drought (Ye et al., 2016). Our work extends these findings by demonstrating that the combination of the two agents achieves greater protection than either alone, likely because *T. asperellum* enhances root nutrient uptake and hormone balance while melatonin directly protects the photosynthetic electron transport chain (Basharat et al., 2025; Wu et al., 2022; Zhou et al., 2016). Crucially, *COMT1* silencing largely compromised *T. asperellum*-induced photosynthetic recovery (**Figure 7**), indicating that endogenous melatonin is essential for maintaining chloroplast function during pathogen challenge. Given that melatonin is known to stabilize photosynthetic complexes and reduce electron transport chain-derived ROS (Ahammed et al., 2024), our data suggest that *T. asperellum* may exert its beneficial effects on photosynthesis at least partly through upregulation of host melatonin synthesis.

**Figure 8:**
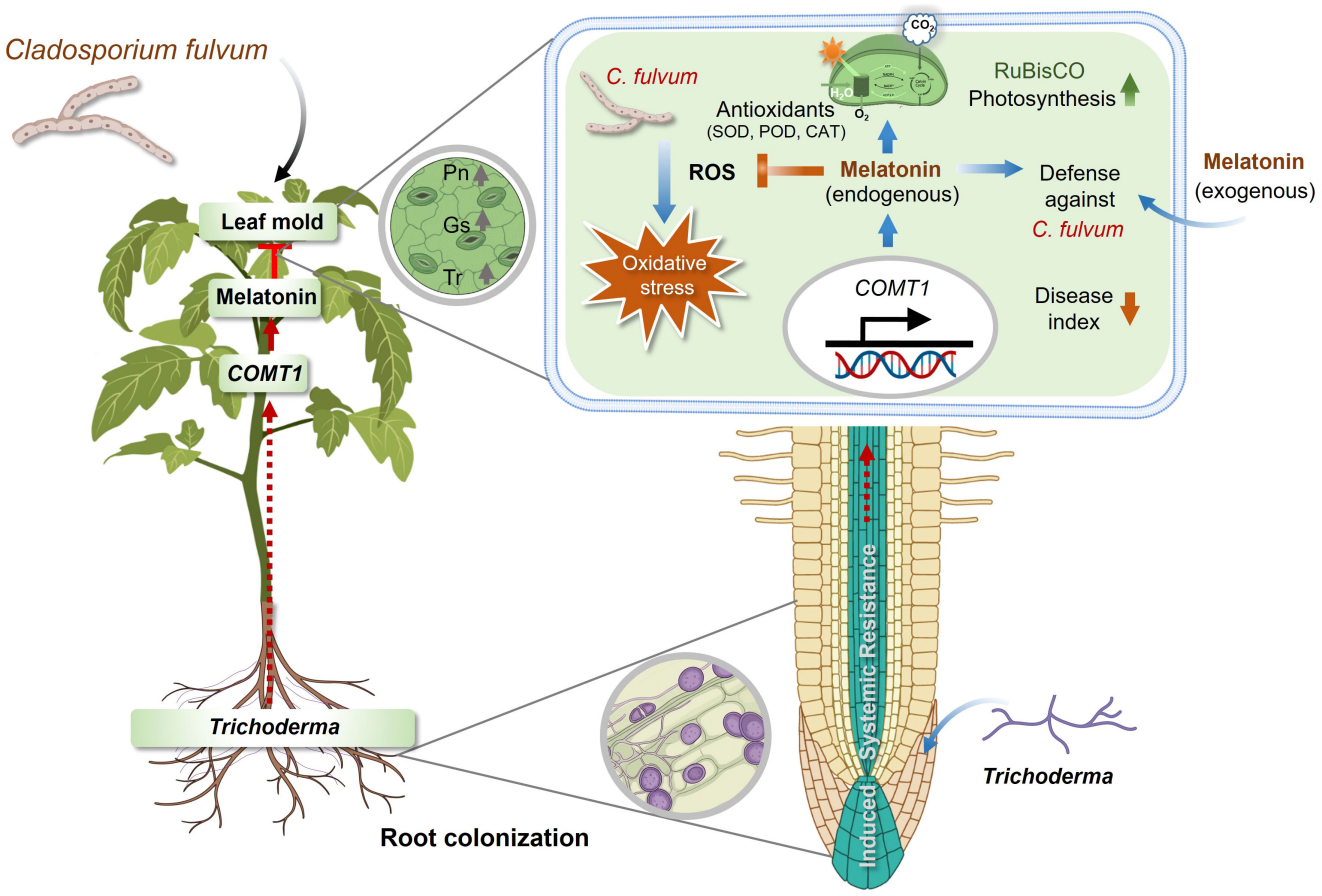
A conceptual model illustrates the mechanism by which *Trichoderma* colonization induces systemic defenses via *COMT1*-dependent melatonin biosynthesis in tomato plants, thereby restoring photosynthetic function and enhancing tomato resistance to leaf mold (*C. fulvum*).

In summary, this study provides compelling evidence that the combination of *T. asperellum* and melatonin synergistically protects tomato against *C. fulvum* by mitigating oxidative stress, boosting antioxidant enzymes, and preserving photosynthetic function. Moreover, we have found that *COMT1*-dependent endogenous melatonin synthesis is essential for *T. asperellum*-induced resistance, although partial compensation by other pathways cannot be ruled out. Taken together, our results support a model in which *Trichoderma* colonization primes systemic defenses, melatonin biosynthesis mitigates oxidative stress, and both converge to restore photosynthetic function, thereby enhancing tomato resistance to leaf mold (**Figure 8**). These findings have practical implications for sustainable agriculture. The integrated use of *T. asperellum* and melatonin could reduce reliance on chemical fungicides, lower environmental pollution, and contribute to integrated pest management in greenhouse tomato production. Future research should further elucidate the molecular signals by which *T. asperellum* activates *COMT1* expression and validate the field efficacy of this combined approach. Nonetheless, our work provides a strong foundation for developing *T. asperellum*-melatonin biopreparations as an eco-friendly tool to control tomato leaf mold and potentially other foliar diseases of solanaceous crops.

## Data Availability

The raw data supporting the conclusions of this article will be made available by the authors, without undue reservation.
